# Medication risk checklist for older adults (LOTTA) – development and validation of a self-assessment tool

**DOI:** 10.1080/07853890.2023.2287707

**Published:** 2023-11-30

**Authors:** Maarit Dimitrow, Roosa Saarenmaa, Marja Airaksinen, Ghada Hassan, Emmi Puumalainen, Markéta Pitrová, Sirkka-Liisa Kivelä, Daniela Fialová, Juha Puustinen, Terhi Toivo

**Affiliations:** a Division of Pharmacology and Pharmacotherapy, Faculty of Pharmacy, University of Helsinki, Helsinki, Finland; b Division of Pharmaceutical Chemistry and Technology, Faculty of Pharmacy, University of Helsinki, Helsinki, Finland; c Department of Social and Clinical Pharmacy, Faculty of Pharmacy in Hradec Králové, Charles University, Prague, Czech Republic; d Clinical Pharmacy Department, University Hospital Královské Vinohrady, Prague, Czech Republic; e Faculty of Medicine, Department of Clinical Medicine, Unit of Family Medicine, University of Turku and Turku University Hospital, Turku, Finland; f Department of Geriatrics and Gerontology, 1st Faculty of Medicine, Charles University and General University Hospital, Prague, Czech Republic; g Service Unit of Neurology, Satasairaala Central Hospital, Wellbeing County of Satakunta, Pori, Finland

**Keywords:** Medication therapy, self-management, risk screening, primary care, patient safety, older adults

## Abstract

**Background:**

Patient safety strategies highlight patients’ own active involvement in ensuring medication safety. A prerequisite for involving patients in their medication therapy is having tools that can assist them in ensuring safe medicine use. Older home-dwelling adults with multiple medications are at high risk for medication-related problems, yet only a few age-specific patient self-administered medication risk screening tools exist. This study aimed to develop, validate, and assess the feasibility of a self-administered medication risk checklist for home-dwelling older adults ≥65 years.

**Materials and methods:**

The draft checklist was formed based on a validated practical nurse-administered Drug Related Problem Risk Assessment Tool supplemented with findings from two systematic literature reviews. The content validity of the draft checklist was determined by a three-round Delphi survey with a panel of 19 experts in geriatric care and pharmacotherapy. An agreement of ≥80% was required. A feasibility assessment (i.e. understandability of the items, fill-out time of the checklist) of the content-validated checklist was conducted among older adults ≥65 years (*n* = 87) visiting community pharmacies (*n* = 4). Data were analysed using qualitative content analysis.

**Results:**

The final validated and feasibility-tested Medication Risk Checklist (LOTTA) for home-dwelling older adults consists of eight items screening the highest priority systemic risks (three items), potentially drug-induced symptoms (one item), adherence, and self-management problems (four items). The checklist proved feasible for self-administration, the mean fill-out time being 6.1 min.

**Conclusions:**

A wide range of potential medication risks related to the medication use process can be identified by patient self-assessment. Screening tools such as LOTTA can enhance early detection of potential medication risks and risk communication between older adults and their healthcare providers. A wider and more integrated use of the checklist could be facilitated by making it electronically available as part of the patient information systems.

## KEY MESSAGES

- Patient safety strategies highlight patients’ own active involvement in ensuring medication safety, which in turn, requires easy-to-use tools to self-assess potential medication risks and communicate them with healthcare providers.
- This study produced a short, age-specific eight item Medication Risk Checklist (LOTTA) to be self-administered by home-dwelling older adults to identify major systemic risks, potential drug-induced symptoms, adherence, and self-management problems related to medication taking.
- To facilitate the use of the checklist in early detection of potential medication risks, future studies should focus on converting the LOTTA list into electronic form and pilot its use as an integrated part of the electronic patient information system.

## Introduction

Both national and international patient safety strategies highlight the role of patients in ensuring their safe care [1,2]. Therefore, healthcare professionals are encouraged to empower patients and/or their carers to be actively involved in care decisions and self-management of their diseases [1,3]. However, to be involved in care decision-making, people need reliable information, suitable tools and resources that support their self-management and cooperation with healthcare providers [1].

The majority of older adults live independently at home and self-manage their diseases, including medications [4,5]. A growing number of older home-dwelling adults have so poor health statuses and functional abilities [6,7] that they may need help from family caregivers or home care personnel to manage their medications safely [8]. Medication self-management at home can pose several risks that can compromise the expected therapeutic outcomes of the medication and can even lead to severe preventable harm [9,10]. This situation calls for easy-to-use, self-administered screening tools focusing on key safety risks of medication therapy for older adults to identify and manage the risks as early as possible.

Existing age-specific medication risk assessment tools are mostly intended to be used by healthcare professionals in various contexts, while some are outdated [11–18]. Our systematic literature searches covering a long period from 1985 up to 2018 found a few patient self-administered medication risk screening tools from the scientific literature [19–23]. Of them, only one is specially designed for home-dwelling older adults [23]. This tool was published in 2018 to be used in the US health system. It focuses on low and high-severity medication risks, and it is intended to be used by people aged ≥55 years. This study aimed to develop, validate and feasibility assess another, a more condensed self-assessment checklist focusing on the highest priority medication risks in home-dwelling older adults.

## Materials and methods

**Figure 1.**
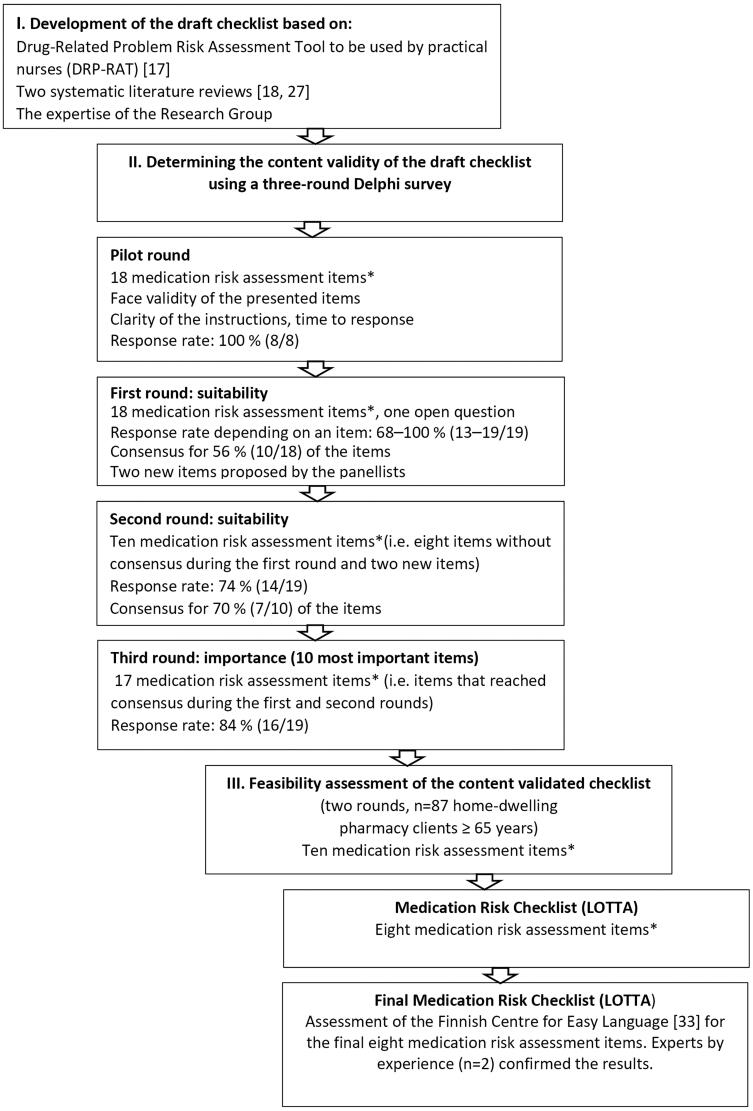
The development process of the medication risk checklist (LOTTA) for older adults. *) multicomponent items are denoted as one item.

This study was conducted using the Delphi consensus method [24]. The development and validation of the medication risk checklist were carried out in three main phases: 1) development of the draft checklist; 2) determination of the content validity of the draft checklist using a three-round Delphi consensus survey; and 3) feasibility assessment of the content-validated checklist among home-dwelling older adults (Figure 1).

Five researchers (MA, MD, TT, RS, GH) formed the steering group that coordinated all phases of the research process, including the development of the draft checklist and carrying out the Delphi rounds. The steering group had regular meetings throughout the study in various stages of the research process.

### Development of the draft checklist

A starting point for the development of the medication risk self-assessment checklist was the structure and content of an age-specific, validated Drug Related Problem Risk Assessment Tool (DRP-RAT) designed to be used by practical nurses in home care [17,25,26] (Figure 1). The DRP-RAT tool was designed to help practical nurses identify the highest priority systemic risks related to the medication use process, potentially drug-induced symptoms (adverse effects), and adherence and self-management problems in medications of older home care clients. The DRP-RAT was also designed to help to find solutions to manage the identified risks. In addition to the experiences with the DRP-RAT tool, a systematic literature review covering January 1, 1985, to April 7, 2016, was conducted focusing on analysing the contents and validation methods and end users of medication risk screening tools for older adults ≥ 60 years [18]. Later, this systematic review was supplemented from April 8, 2016, to December 10, 2018, with another systematic literature review conducted in the Finnish-Czech collaboration [27]. The clinical expertise of the research group was used throughout the development process.

For the draft checklist, by joint decision of the steering group, items from the DRP-RAT tool [17,25,26] were selected that 1) were considered suitable for being self-assessed by home-dwelling older adults themselves without assistance, and 2) were relevant to be assessed when the medicines were self-managed at home (e.g. whether the person had an up-to-date medication list and whether he/she had experienced symptoms that were potentially drug-induced). Brief clarifying comments were added to make it easier for older adults to self-assess the items (e.g. what is meant by the medication list and which medicines should be included). Some of the DRP-RAT items were also divided into two separate items. In this phase, also research evidence systematically gathered from the literature was used to guide decision-making of the steering group.

Three researchers (EP, MD, MA) read and analysed the literature found in the first systematic literature search [18] and five researchers (MP, EP, MD, MA, TT) read and analysed the literature found in the supplementing systematic literature search [27]. The draft checklist derived from DRP-RAT tool [17,25,26] was supplemented with the findings of the systematic literature reviews [18,27]. They provided an understanding of the most common prognostic indicators for medication safety risks included in the risk management tools. Based on this evidence, the draft checklist was complemented with new items not found in the DRP-RAT tool, such as difficulties in using medical devices, several changes in the medication regimen, and lack of regular therapeutic outcome monitoring. Finally, the steering group decided on the new items to be included in the draft checklist for the experts to review in Delphi rounds. Because the checklist was meant to be self-administered by older adults, special attention was paid to the structure (as simple and brief as possible), language (lay language terms) and understandability of the items of the draft checklist to older adults.

### Delphi method: selection of expert panellists

The Delphi method was applied in this study as it is widely used to gain expert consensus on a chosen topic with incomplete or missing information and shared understanding [24,28]. The method is widely applied in health care, also in medication risk management [17,24,29–31]. This study involved three Delphi rounds online *via* eDelfoi software [32]. Each round was conducted within one week between May and June 2019 (Figure 1). Altogether 30 experts were invited to participate in the survey, and 19 of them accepted the invitation (8 physicians, 11 pharmacists). The selection criterion for the panellists was having clinical experience in geriatric care and pharmacotherapy.

### Delphi rounds for content validation

Before actual Delphi rounds, a pilot round was conducted to assess the clarity of the instructions and face validity of the items of the draft checklist (Figure 1). The pilot round also included an open-ended question *‘If you think that the draft checklist ignores some essential aspects, please propose each of them’*. A separate expert group was invited for the pilot round. It consisted of 8 experts (one physician and seven pharmacists, all having expertise in geriatric care and pharmacotherapy) of the invited 11 persons. The pilot panellists’ comments and proposals led to few modifications in the final draft checklist before sending it to the actual Delphi panel.

One of the authors (MD) first analysed each Delphi round’s (including the pilot round) responses. The results were then discussed in the steering group meetings between the rounds. The final form of the next round’s items was decided based on these discussions.

Before each Delphi round, the panellists were emphasised to consider the intended use of the checklist, focus on the most important medication risks of home-dwelling older adults ≥ 65 years, and how the checklist could be self-administered by older adults or their proxies. Thus, the items should be easy to understand and not cause additional concern.

During the first and the second Delphi rounds, the panellists were instructed to rate the suitability of each item of the draft checklist in assessing medication risks for home-dwelling older people ≥ 65 years. They were also asked to answer the following open question during the round one: *‘If you think that the draft checklist is missing some essential aspects, please propose each of them’*.

The second round included items for which consensus was not reached during the first round. They were modified according to the panellists’ recommendations. New items were included according to the panellists’ proposals. Panellists also were informed about the items that reached consensus during the first round.

The first and second Delphi rounds produced 17 items altogether: during the first round, the consensus was reached for ten, and during the second round, for seven items (multicomponent items are denoted as one item) (Figure 1). As the final checklist was intended to be as simple and brief as possible, focusing on the most important medication risks, during the third round, the panellists were asked to rate the ten most important items to be included in the final checklist. Likewise, they were asked to rate the ten most significant potentially drug-induced symptoms to be included in the final checklist (assuming that the item assessing potentially drug-induced symptoms was rated by the panellists to be included in the checklist).

### Data analysis

A 4-point Likert scale (3 = suitable, 2 = suitable if modified, 2 = not suitable/not necessary, 0 = I cannot say) was applied to rate the suitability of the items and sub-items. Items that ≥80% of the panellists rated suitable for self-assessing medication risks of home-dwelling older people ≥65 years were included in the checklist. The number of panellists’ ratings determined the importance of the items. The results are reported in frequencies and percentages.

### Feasibility study

Feasibility, i.e. understandability and fill-out time of the Delphi panel validated Medication Risk Checklist (Figure 1), was assessed by older home-dwelling adults visiting any of the four community pharmacies participating in the study. The inclusion criteria for the feasibility study were: being a client at one of the pharmacies, having at least one of her/his own medicines dispensed from the pharmacy, age ≥ 65 years, and being able to self-administer the items of the checklist independently without anyone’s help. The study pharmacies were located in different parts of Finland (capital area and counties) and were selected as a convenience sample.

The feasibility study included two rounds (Figure 1). Before the first round, one of the researchers (RS) trained the pharmacists in participating community pharmacies to know the purpose and use of the checklist and the conduction of the feasibility study. After the preparatory phase, the pharmacists started to invite clients ≥ 65 years old to participate in the study. The goal was to get a total of 50–100 older home-dwelling pharmacy clients to complete the checklist. The consented participants self-completed the checklist anonymously and independently at the pharmacy. The researcher (RS) registered the time spent completing the checklist using a watch. After completing the checklist, the researcher (RS) interviewed each study participant about the understandability of the checklist items and how they felt about completing the checklist. The questions asked were: (1) ‘How did you feel about answering the checklist items?’ (2) ‘Did you understand what the items mean?’ and (3) ‘If an item was difficult to understand, how it should be modified to be more understandable?’

The answers to the questions and the time spent completing the checklist were documented on a separate paper form, together with other comments and the respondent’s age. If the participants had questions about their medicines use during or after self-completing the checklist, they were discussed in a usual manner with a pharmacist in the community pharmacy.

The results were analysed using qualitative content analysis, and the checklist items were modified based on the results. The modified checklist was tested in the second round of the feasibility study in one of the four community pharmacies.

To ensure that the language used in the final feasibility tested Medication Risk Checklist is easy to understand, the Finnish Centre for Easy Language (Selkokeskus) [33] assessed the language (Figure 1). The Centre promotes communication, information, and culture in the easy Finnish language. Finally, experts by experience (*n* = 2) evaluated the understandability of the items assessed by the Finnish Centre for Easy Language (Selkokeskus) [33].

### Research ethics

According to the research ethics guidelines in Finland an ethics committee pre-evaluation of the study protocol was not required because the study did not contain any medical intervention to patients and no patient-specific health data was collected for the study [34]. The data that was collected was anonymous and related to the understandability of the items on the checklist and the time spent filling out the items. Filling out the checklist was voluntary. If potential medication risks or questions about medication therapy arose due to filling out the checklist, the pharmacists at the community pharmacy managed them as their usual practice. The data security and the privacy protection of the participants were ensured throughout the whole study according to the European Union’s General Data Protection Regulation and the data security legislation of Finland. Written research permissions were obtained from each participating pharmacy.

## Results

### Delphi study

Altogether 19 expert panellists (8 physicians, 11 pharmacists) participated in the Delphi rounds. They had wide experience in geriatric pharmacotherapy. The physicians’ expertise covered geriatrics, internal medicine, general practice, neurology and clinical pharmacology. Seven of them had higher academic degrees (PhDs). Of the pharmacists, five were accredited in comprehensive medication review, two had medication review competence, and one was also a practical nurse. Three of the pharmacists had PhD degrees.

The response rate during the Delphi rounds varied from round to round. During the first round, the response rate varied from 68% to 100% (*n* = 13–19/19) depending on an item. During the second round, the response rate was 74% (*n* = 14/19), and during the third round, 84% (*n* = 16/19) (Figure 1).

### Importance rating

During the first and second Delphi rounds, the panellists reached consensus for 17 medication risk screening items suitable for being self-assessed by older adults. Of those, they ranked the ten most important items with sub-items during the third Delphi round (Table 1).

**Table 1.** The ten most important medication risk assessment items rated by the panellists (*n* = 16) during the third Delphi round.

Item/subitem	Number of ratings for each item/sub-item n(%) n(%)	
1. Do you have a medication list containing all medicines that you use, with doses? The list should contain all prescription medicines, over-the-counter (OTC) medicines, vitamins, minerals, and natural and herbal products that you are using.		15 (94)
2. In the last four weeks, have you had any of the following symptoms repeatedly interfering with your everyday life?		12 (75)
*a) Feeling unusual tiredness or drowsiness in the daytime*	*14(88)*	
*b) Dizziness*	*14(88)*	
*c) Falls*	*13(81)*	
*d) Unusual dryness of the mouth*	*12(75)*	
*e) Constipation or other gastrointestinal problems*	*12(75)*	
*f) Confusion*	*11(69)*	
*g) Urinary problems (urinary incontinence or difficulty with urination)*	*9(56)*	
*h) Bruising easily or getting nosebleeds easily*	*9(56)*	
*i) Nausea*	*8(50)*	
*j) Memory problems*	*8(50)*	
3. Do you have problems or is it unclear to a) remember to take your medicines on time, b) take medicines according to the instructions, c) know the purpose of the medicine, d) know how long period you take medicine, e) know the goals of your medication treatment, f) follow/monitor the effects of medication treatment, g) administer the medicines (e.g. to drop the eye-drops, to dose asthma medicines, to inject the injectable medicines or to open medicine packages), h) halve the tablets, i) swallow tablets or capsules?		12(75)
4. Do you sometimes take on purpose more or fewer medicines than the physician has prescribed or leave the medicine not taken at all?		12(75)
5. Do you have more than one physician involved in your care (e.g. general practitioners, specialists, private practitioners)?		11(69)
6. Do you use some of the following medicines without regular follow-up a) painkillers, b) diuretics, e.g. for hypertension or swellings, c) medicines for lowering high cholesterol (statins), d) anticoagulants (e.g. Marevan*, Xarelto*, Eliquis*), e) antidepressants, f) sedatives or medicines for treating anxiety, g) medicines which your physician is not aware of, h) heartburn or upper stomach disorders medicines (e.g. Somac*, Rennie*, Gaviscon*)?		11(69)
7. Have you continuously used prescribed sleeping pills for over three months (do not apply melatonin)?		10(62)
8. Has a physician or pharmacist, in cooperation with a physician, assessed the appropriateness and necessity of all the medicines you took during the last year?		9(56)
9. Are you sometimes forced to go short of your necessary medicines because of economic problems?		9(56)
10. Have you used any of the following products, without that a physician or pharmacist would have assessed their suitability to your other medicines or your illnesses a) over-the-counter medicines (e.g. painkillers, flu medicines, allergy medicines, heartburn, or constipation medicines), b) vitamins, minerals, herbal products/medicines?		8(50)

* Commonly used product names in Finland, which were used to help the identification of medicines.

The item screening whether an older adult has an up-to-date medication list was most often rated among the most important items to be included in the final checklist (94%, *n* = 15/16) (item 1, Table 1). The panellists also reached consensus of the importance of the items screening: potential drug-induced symptoms (e.g. potential adverse effects caused by medicines); poor medication self-management (e.g. having problems in administering the medicines); and poor medication adherence (items 2–4, Table 1), each item rated among the most important ones by 75% (*n* = 12/16) of the panellists. Of the potential drug-induced symptoms: feeling unusual tiredness or drowsiness in the daytime, and dizziness (both 88%, *n* = 14/16), and falls (81%, *n* = 13/16) were most often rated among most important by the panellists and, thus considered among the most important sub-items to be included in the final checklist. Systemic risks like having several physicians involved in older adult’s care and lack of therapeutic outcome monitoring (items 5 and 6, Table 1) were rated the most important by 69% (*n* = 11/16) of the panellists.

### Feasibility study

Feasibility study focused on the ten most important items with sub-items rated by the panellists during the third Delphi round. Altogether 87 pharmacy clients ≥65 years (range 65–86 years) participated in the feasibility study.

A total of 83 older pharmacy clients visiting any of the four participating community pharmacies (P1-P4) self-administered the checklist during the first round of the feasibility study. Three of those were rejected (P4) as they did not meet the inclusion criteria (one client was 61 years old, two checklists were completed by a caregiver). Thus, from the first round, altogether 80 pharmacy clients (P1: *n* = 10, P2: *n* = 23, P3: *n* = 23, and P4: *n* = 24) were included in the feasibility study consisting of self-administering of the checklist, followed by a brief interview by a researcher. From the second round that tested self-administering of the revised checklist, followed by a brief interview by a researcher, all clients (*n* = 7) were included in the study. The second round was carried out in one of the pharmacies (P3).

The mean age of the participants (*n* = 80) in the first round was 74 years. It took on the average of 6.5 minutes to self-complete the checklist. Considering the checklist’s understandability, the most important result was that 22% (*n* = 18/80) of the respondents did not understand what a medication list means (Table 2). Furthermore, items related to medication review and appropriate follow-up/monitoring of medication therapy were difficult to understand and self-assess.

**Table 2.** Key findings of the first round of the feasibility study.

Items(s) or themes of the content validated checklist	Feasibility	Modifications in the final checklist
Do you have a medication list containing all medicines and their doses that you use? The list should contain all prescription medicines, over-the-counter (OTC) medicines, vitamins, minerals, and herbal products that you are using (Table 1, item 1).	22 % (*n* = 18/80) of the respondents did not understand what was meant by a medication list	The item was modified to be more concrete. It was added that the list could be a paper or electronic listing of the medicines in use.
Items related to the use of potentially inappropriate medicines (PIM) or the use of certain medicines (e.g. diuretics, statins, anticoagulants, sleeping pills) without proper follow-up (Table 1; items 6, 7 and 10).	Respondents had difficulties answering whether they used medicines mentioned in the preliminary list. Furthermore, they needed help answering how the follow-up of medication therapy had been carried out.	Items related to the use of certain PIMs or follow-up of certain medicines were excluded. Instead, PIM use was assessed with an item of potential adverse effects of these medicines. Items related to the follow-up of certain medication therapy were modified to a more general form.
Item related to medication review (Table 1, item 8).	Item was too difficult to understand. Of the respondents, 6.5 % (*n* = 5) did not understand what was meant by medication review.	The item was removed because the respondents did not know how to answer it. They did not know what the medication review meant and whether such had been done. Other items on the checklist indicate whether there is a need for a medication review.
Items were added based on the findings of the feasibility study, recommendations of the research group, and published literature.	Items were tested in the second round, and they were understandable.	The added items: Do you feel that the medicine prescribed by your physician is not suitable for you? Do you have to compromise on necessary medicines because you can’t afford to buy them?

Based on the qualitative analysis of the results and the expertise of the research group, some items were modified or totally removed from the final checklist (Table 2). In addition, based on published literature, the research group decided to add two new items assessing older adults’ own perceptions about their medication (Table 2). The modified checklist was tested in the second round (7 respondents, mean age 75.0 years). The results of the second round showed that the checklist was understandable, and the time to fill out the checklist was 6.1 min. Furthermore, experts by experience (*n* = 2) confirmed the results.

### The final medication risk checklist (LOTTA)

The feasibility study resulted in an eight-item Medication Risk Checklist that was named LOTTA (Table 3). The final checklist was divided into three main sections: 1) Systemic risks (items 1–3), 2) Potentially drug-induced symptoms (item 4), and 3) Medication adherence and self-management (items 5–8). All the items on the checklist have the same options for expressing the self-estimate: ‘yes’, ‘no’, and ‘I can’t say’. The items are modified so that in each item, the answer ‘yes’ or ‘I can’t say’ indicate risk (Table 3). The language used in the checklist met the criteria of Easy Language (Finnish), and the SELKO symbol was achieved [33].

**Table 3.** The final medication risk checklist (LOTTA) (assessed by the Finnish Centre for easy language (Selkokeskus) [33]).

Please answer the items below. Tick the box next to the option that best describes your situation. If you answer "yes" or "I can’t say" in multiple items, please contact your pharmacy, physician, or nurse. Also, contact them if you have any questions when completing the checklist.			
1. Are you missing an up-to-date paper-based or electronic medication list containing all the medicines that you are using at the moment? Such a list should contain all prescription medicines, over-the-counter (OTC) medicines, vitamins, minerals, and natural and herbal products that you are using. Yes □ No□ I can’t say □			
2. Do you have more than one physician involved in your care (e.g. general practitioner, specialist, private practitioner)? Yes □ No□ I can’t say □			
3. Are you lacking regular follow-up of your medication therapy (e.g. control visits in health care or home-based testing/measurements)? Yes □ No□ I can’t say □			
4. In the last four weeks, have you had any of the following symptoms repeatedly interfering with your everyday life?			
	Yes	No	I can’t say
• Feeling unusual tiredness or drowsiness in the daytime	□	□	□
• Dizziness	□	□	□
• Falls	□	□	□
• Urinary problems (urinary incontinence or difficulty with urination)	□	□	□
• Nausea	□	□	□
• Constipation or other gastrointestinal problems	□	□	□
• Memory problems	□	□	□
• Confusion	□	□	□
• Bruising easily or getting nosebleeds easily	□	□	□
• Unusual dryness of the mouth	□	□	□
5. Is it unclear to you for how long period you need to be taking your medicines? I.e. Are your medicines intended for regular use, for periodic courses, or are they to be taken ‘as needed’? Yes □ No □ I can’t say □			
6. Have you had the following issues when using your medicines? a. You don’t know what exactly your medicines are for Yes □ No□ I can’t say □ b. You have difficulties in taking your medicines as instructed, e.g. You don’t understand the dosing instructions You don’t remember to take the medicines when instructed Taking medicines does not fit in with the schedule of your daily activities Yes □ No□ I can’t say □ c. You have difficulties in administering the medicines, e.g. You don’t know how to dose your eye drops, or it is difficult for you to dose eye drops. You don’t know how to dose your asthma medicines, or it is difficult for you to dose asthma medicines You don’t know how to inject your medicines yourself, or it is difficult for you to inject your medicines You have difficulties opening medicine bottles or packaging You have difficulties in halving tablets Yes □ No□ I can’t say □ d. You have difficulties swallowing tablets or capsules Yes □ No□ I can’t say □			
7. Do you feel that any of the medicines prescribed to you is not suitable for you? Yes □ No□ I can’t say □			
8. Are you sometimes forced not to get essential medicines for your care because of financial problems? Yes □ No□ I can’t say □			

### Section 1: Systemic risks (items 1–3, Table 3)

The first section of the Medication Risk Checklist (LOTTA) includes the highest priority systemic risks related to the medication use process, such as lack of an up-to-date medication list, several physicians involved in older adult’s care and lack of therapeutic outcome monitoring. These items may indicate fragmentation of the patient’s medication use process and thus, pose medication risks.

### Section 2: Potentially dug-induced symptoms (item 4, Table 3)

The second section of the LOTTA-checklist focuses on pharmacotherapy related risks. This section lists symptoms potentially caused by medicines commonly used by older adults, repeatedly interfering with everyday life. The ten drug-induced symptoms listed in the checklist cover potentially harmful symptoms or adverse effects caused by potentially inappropriate medicines for older adults (e.g. anticholinergics, sedatives, neuroleptics), but also other medicines commonly used by older adults in clinical practice (e.g. diuretics, antihypertensives, antidiabetic medicines).

### Section 3: Medication adherence and self-management (items 5–8, Table 3)

Section 3 concentrates on items indicating potential risks related to not taking the medicines as prescribed or having problems in self-management of the medication use at home. This section also contain items assessing perceived medication-related burden and experiences with medicines, e.g. has the person a feeling that any of the medicines prescribed to her/him is not suitable for her/him or the medicine taking does not fit in with the schedule of daily activities.

## Discussion

Development of the Medication Risk Checklist (LOTTA) was a rigorously selective process which produced a content validated and feasibility tested medication risk checklist that can be self-administered by older adults to screen medication risks at home. The brief, one-page checklist, also assessed for being easy-to-read for older adults can be filled in about 6 minutes providing a multi-faceted understanding of the highest priority systemic risks related to the medication use process, potential drug-induced symptoms (adverse effects) caused by medicines commonly used by older adults, and problems in adherence and medication self-management.

It is surprising, that although most of the medicines are used at home and older adults are the major user group [35,36], only a few age-specific, self-administered medication risk screening tools are designed for them. Our comprehensive systematic reviews covering the research literature until December 10, 2018 [18,27] found only one validated and published medication risk checklist specially targeted to home-dwelling older adults and designed to be self-administered by them [23]. This checklist originates from the United States and was published in 2018. This 24-item ‘MedUseQ’ tool with a full name ‘A Self-Administered Screener for Older Adults to Assess Medication Use Problems’ focuses on low and high-priority patient and medication-related risk factors associated with medication nonadherence. The difference from the LOTTA Checklist is that the MedUseQ tool does not include systemic factors predisposing to medication risks. Like the LOTTA Checklist, the MedUseQ tool contains items assessing patient experiences of medicine taking, such as medication-related burden [37], as potential risk factors.

To cover the most recent literature on medication risk self-assessment tools for home-dwelling older adults, we updated the systematic literature search for the period from Jan 1, 2018, to Oct 27, 2023. The new search found five publications [38–42] describing five medication risk assessment tools, three of which [38,39,42] were intended to be patient self-administered. Of them, two tools [38,39] were developed for hospital setting and one for primary care [42]. However, the tool developed for primary care [42] did not meet our inclusion criteria (i.e. a self-administered medication risk assessment tool for home-dwelling older adults ≥ 65 years). The tool was targeted for home-dwelling adults ≥ 18 years with polypharmacy and was designed to be filled in before general practitioner (GP) consultation to encourage patients with polypharmacy to be involved in medicines optimisation. Thus, it seems that no new age-specific self-administered medication risk assessment tools for home-dwelling older adults has been published since the development of the LOTTA Checklist, making LOTTA Checklist still timely and needed. The updated literature search was conducted using the same databases (Medline Ovid, Scopus, Web of Science (WOS), Evidence-based Medicine (EBM)) and the same search strategies were used as in the previous systematic searches by Puumalainen et al. 2019 [18] and Pitrová 2021 [27].

Among the unique features of the LOTTA Checklist compared to previous patient self-administered medication risk screening tools [19–23] is inclusion of items assessing systemic risks. None of the previous tools that we found had included such common systemic risk factor as lack of up-to-date medication list in the self-assessment checklist. Lack of reconciled medication list is a common challenge globally jeopardising patient and medication safety [1,43]. If included in the checklist, the checklist can perform as an awareness raiser of the need for an up-to-date medication list among older adults. E.g. in Finland, all prescriptions are currently issued and dispensed electronically *via* a national patient information database Kanta, but the system does not produce an up-to-date medication list for medicine users [44,45]. According to a recent population-based survey in Finland by the National Medicines Agency, only 20% of the respondents reported having an up-to-date medication list [46]. Similar results were obtained from a recent regional health screening study among 75-year-old home-dwelling residents (*n* = 953), which used the LOTTA Checklist as an outcome indicator for medication-related risks [47]. Nearly half (43%) of the older medicine users missed an up-to-date medication list. The feasibility part of our study showed that 22% of the participating home-dwelling older people (*n* = 80) visiting the community pharmacy did not even understand what was meant by a medication list.

The LOTTA checklist may help older adults also to identify potentially drug-induced symptoms, i.e. adverse effects, and when they should contact their health care providers because of them. This feature is important as drug-induced symptoms are difficult to identify by older adults and thus tend to be underreported and ignored, not properly managed as part of the normal care process [48]. Based on the literature and DRP-RAT tool [17,25,26], we created a large list of symptoms that could be potentially caused by medicines commonly used by older adults that was submitted to our expert panel for evaluation. The majority (8 out of 10) of the ten most important drug-induced symptoms produced by the Delphi study and listed in the LOTTA Checklist are the same as listed in the practical nurse-administered DRP-RAT tool [17,25,26]. Only two symptom items listed in the LOTTA checklist, i.e. ‘Bruising easily or getting nosebleeds easily’ and ‘Unusual dryness of the mouth’, were not listed in the DRP-RAT tool. However, those symptoms are quite common [47] and may significantly impair the quality of life and functional ability. The LOTTA Checklist was utilised as a part of a comprehensive health screening for 953 home-dwelling 75 year old residents in the PORI75 epidemiological study [47]. The participants frequently self-reported symptoms listed in the checklist, the most prevalent symptoms being constipation (21%), urinating problems (20%), unusual tiredness (17%), bruising easily or getting nosebleeds easily and unusual dryness of the mouth (both 16%). These results confirm the validity of the listed drug-induced symptoms in the LOTTA Checklist.

### Potential practical implications

Different ways of utilising and applying the LOTTA Checklist have emerged since it was launched in Finland in 2020, after its validation process and feasibility testing were completed. The checklist and instructions for its use for older adults and health care professionals are freely available in printable and electronic version from the webpages of the Finnish Medicines Agency and the Association of Finnish Pharmacies [49,50]. Furthermore, many community pharmacies provide the list for their older clients. When using the checklist, older adults are encouraged to list their medicines in use or ask for an up-to-date medication list from health care professionals.

The Medication Risk Checklist (LOTTA) can also be used in assessing the need for different social or healthcare services. This way of use has been piloted in a large epidemiological cohort study which utilised the checklist as a part of comprehensive health screening among older adults aged 75 years [47]. The study gathered information on medication-related risk factors among home-dwelling older adults to be used in future planning and implementing health services. Another recent study utilised the checklist when piloting a remote pharmacist service for multi-professional municipal case management for home-dwelling older adults [51]. The pharmacy service included remote medication review using the LOTTA Checklist to screen potential medication risks in clients’ medication and to assess the need for a multi-professional comprehensive medication review. The results of this study indicate that by using the checklist, it was possible to identify medication risks.

The list could be promoted by incorporating it in the electronic medication use process, and online care services for medicine users. This way, the older adults could complete the list independently at home, and healthcare professionals would easily access information on potential medication risks. The checklist can also be used as an electronic indicator providing useful information for planning health services in the future [47]. Health and social care personnel in health centres can use the checklist to identify older risk patients, e.g. when conducting health checks.

Furthermore, the LOTTA Checklist can perform as a communication tool between the patient and healthcare. If the list has been filled in advance at home, the results can be reviewed together with a physician or nurse at an appointment. This information can facilitate discussion on a patient’s medication therapy. The checklist is suitable to be filled in and also discussed in community pharmacies. It can be utilised to identify older adults with potential medication risks, and pharmacy professionals can guide those for interventions, e.g. medication reviews. Pharmacies also can utilise the checklist as an interview tool during medication counselling.

As described above, the LOTTA Checklist may enable the identification and solving of medication risks at an early stage in primary health care. This point is important as medication-related problems consume healthcare resources by causing, e.g. emergency department visits in hospitals, an identified challenge in geriatric care [52,53]. Furthermore, identification and solving of medication-related risks as early as possible can potentially support the independence and participation of older adults in their pharmacotherapy and living independently at home.

### Strengths and limitations of the study

The use of the Delphi method in validating the content of the checklist is justified as 1) there is a lack of published guidance for this kind of screening criteria, and 2) the method has widely been applied in developing different screening criteria for use in health care [17,24,29–31].

An interprofessional expert panel of 14–19/19 members (physicians and pharmacists) responded in three Delphi rounds (Figure 1). Akins and colleagues (2005) [54] found in their study that reliable outcomes in the Delphi survey can be obtained with a relatively small expert panel, provided that the experts are carefully selected. Thus, we believe that in this study, the number of experts (selected with strict inclusion criteria) was high enough to produce reliable outcomes. It is also established that 2–3 Delphi rounds can be considered sufficient to obtain reliable results [55]. This study applied a 4-point Likert scale and *a* ≥ 80% limit of consensus, which we believe is sufficient to produce good reproducibility. In Lange’s study (2020) [56], test-retest reliability varied considerably depending on the rating scale used (3, 5 or 9-point scale). The best reproducibility was produced on a three-step scale. The differences between the scales increased as the consensus limit increased. Response rates in the Delphi rounds in the current study were quite high (74–100%), indicating the panellists’ involvement and commitment to the study.

Although the draft checklist was based on two systematic literature reviews [18,27], and an existing risk-assessment tool DRP-RAT [17,25,26], it may have missed some relevant aspects of medication risk screening. Because of this, we asked the expert panellists during the pilot and the actual rounds to suggest additional items based on their clinical expertise. We also utilised the geriatric expertise of our research group to complement the items. Thus, the development processes of the checklist have been strict enough to tackle potential deficiencies with the methodology.

The literature searches were restricted to English-language articles and did not include grey literature, which may be a limitation as some important articles may have been missed. The draft checklist was also based on an existing risk assessment tool DRP-RAT [17,25,26], which due to the constructive nature of a Delphi survey [24], may be a limitation (possible false conclusions may have remained from one criterion to another). On the other hand, the DRP-RAT tool has been proven to be sensitive in identifying clinically significant DRPs [26,57], which may be considered a strength.

The feasibility of the Medication Risk Checklist (LOTTA) was tested in a real community pharmacy environment with ordinary older adults ≥ 65 years visiting pharmacy. The participating four pharmacies located in different parts of Finland (capital area and counties) with different population bases. The study participants were quite evenly distributed among the participating pharmacies. Thus, it is likely that those older pharmacy clients who participated the feasibility study represented a real-life sample of older adults with different health statuses and different socio demographic backgrounds, which is the strength of this study.

### Future studies

More research is needed to prove the ability of the checklist to identify older people with medication risks and the usability of the checklist in clinical practice. Studies with different healthcare organisations, e.g. health centres or hospitals, could indicate items showing a particularly high risk. It would also be useful to study if the self-administered checklist can empower older adults to keep their medication lists up-to-date and identify and report potential medication therapy problems to healthcare professionals.

## Conclusions

This study developed, validated and assessed the feasibility of an eight-item patient self-administered Medication Risk Checklist (LOTTA). A wide range of potential medication risks related to the medication use process can be identified by patient self-assessment. Screening tools such as LOTTA can enhance early detection of potential medication risks and risk communication between older adults and their healthcare providers. A wider and more integrated use of the checklist could be facilitated by making it electronically available as part of the patient information systems.

## Data Availability

Inquiries regarding the data of this study can be directed to the corresponding author (Maarit Dimitrow).
